# Fertility preservation healthcare circuit and networks in cancer patients worldwide: what are the issues?

**DOI:** 10.1186/s12885-018-4046-x

**Published:** 2018-02-17

**Authors:** Kathleen Melan, Frederic Amant, Jacqueline Veronique-Baudin, Clarisse Joachim, Eustase Janky

**Affiliations:** 1 Laboratory CELTEC Cancer and Environment EA4546, University of the French West-Indies, Pointe-à-Pitre, Guadeloupe; 2 Department of Obstetrics & Gynaecology, UZ Gasthuisberg / Katholieke Universiteit Leuven Herestraat 49, 3000 Leuven, Belgium; 3 Oncology Haematology Urology Pathology Department, UF 1441 Cancer Research and Registry, University Hospital of Martinique, 127 Route de Redoute, Les jardins de la Mouïna, 97200 Fort-de-France, Martinique; 4 Gynaecology, Obstetrics Department, University Hospital of Guadeloupe, Pointe-à-Pitre, Guadeloupe

**Keywords:** Oncofertility, Cancer therapies, Fertility preservation, Quality of life, Healthcare management

## Abstract

**Background:**

Fertility preservation (FP) is a major determinant of quality of life after cancer remission for women who may not have achieved their ideal family size. This article describes the FP services and strategy currently available, highlighting issues of oncofertility worldwide.

**Main body of the abstract:**

For these patients in complex situations, health networks are essential to improve coordination of care, and the strengthening of this coordination is a major challenge to improve the performance of the health system. Two international networks have been created in order to foster scientific exchange between countries and to standardize the oncofertility healthcare circuit. However, the paucity of referral nationwide networks lead to a structural gap in health care policies.

**Short conclusion:**

Management strategies of oncofertility in the world are still fragile and uneven. To structure the oncofertility sector, a multidisciplinary project allowing teams to collaborate is of utmost importance particularly in low and middle-income countries.

## Background

Oncofertility is a new transversal concept that describes an integrated network focused on medical methods to spare or restore reproductive function in patients diagnosed with cancer. The term was coined in 2006 in the USA, although the history of oncofertility dates back to 1971, with the signature of the National Cancer Act. Oncofertility connects disciplines such as oncology, reproductive medicine, sexology, pediatrics and bioethics. Gone are the days when the only goal was to cure cancer, and oncofertility has taken over as the medical field concerned with minimizing the negative effects of cancer treatment on the reproductive system and fertility, and aiming to assist individuals with reproductive impairments resulting from cancer therapy.

The substantial growth of the field of oncofertility is the result of increased cancer incidence and especially increases in post-cancer survival. In 2020, more than 7.9 million women will be diagnosed with cancer worldwide: around 3.7 million in Asia; 1.7 million in Europe; 1.5 million in America; 604,000 in Africa and 50,000 in the Caribbean [1]. Among female cancer survivors, 1 in 250 are of reproductive age [2]. Current therapeutic advances had led to growing numbers of young women who survive their cancer. It is considered that one young adult aged 20 to 30 years out of 1000 has survived a cancer in childhood [3]. In Europe, children diagnosed with cancer currently have a 5-year-survival rate of 79.1% [4]. In the USA, about 10 % of all female cancer survivors are younger than 45 years of age [5] and this rate is around 18.6% in the French West-Indies (FWI, Martinique). Every year, more than 15,000 reproductive-age women in France face a cancer diagnosis.

Both cancer and oncologic treatments are known to induce sexual dysfunction, gonadotoxicity and multiple mechanisms of impaired reproductive function, though the effects may be unpredictable [6–9]. A study conducted in South India by Rajendranath and al [10] on the long-term effects of cancer treatment in childhood cancer survivors found that 24.5% of them were diagnosed with impaired fertility; it was the first long-term effect found. The most constant determinants of fertility disorders in cancer survivors are chemotherapy, radiotherapy or surgery involving the reproductive organs. The available literature quantifying infertility risks has reported the highest risk rates (> 80%) associated with chemotherapy with alkylating agents.

Fertility preservation (FP) is a major determinant of quality of life after cancer remission for women who may not have completed their family or achieved their ideal family size [11].

It is essential to inform cancer patients about new techniques for fertility preservation and to integrate them into systematic long-term follow-up. Young women will have the opportunity to preserve reproductive functions without significant impact on their survival, as a result of this recently defined concept. Advances in oncofertility are the only hope to ensure future fertility of cancer patients worldwide.

Despite advances in technology and knowledge in the field of oncofertility, there is a major gap in the structure of fertility preservation management strategies in the world and in developed countries. This induces a lack of knowledge about fertility management options or more marked inequalities in access to care at referral centers among young women and girls diagnosed with cancer. In order to provide the hope of future fertility and to reduce disparities in access to care among all young female cancer survivors, it is currently important to study how fertility preservation networks are structured for cancer patients. This work aims to outline the landscape of organizational models and the chain of coordination of fertility preservation worldwide for female cancer patients. A literature review was conducted in 2016 by searching the electronic Medline and EMBASE databases for original and review articles concerning “fertility preservation”, “oncofertility network” and “fertility after cancer” published up to 1st September 2016.

## Main text

### Fertility preservation healthcare circuit for young women: current strategy

**Fig. 1:**
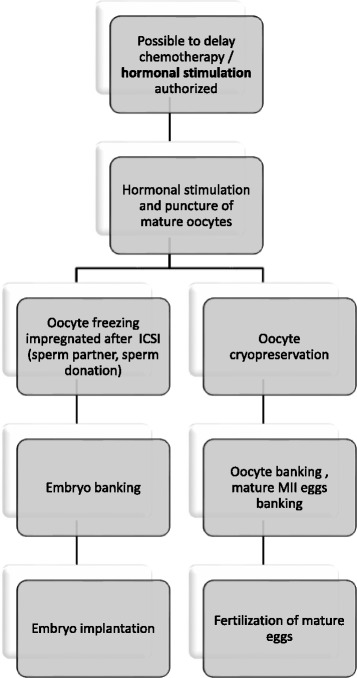
Current therapeutic strategy for female oncofertility when it is possible to delay oncotherapy. ICSI: Intracytoplasmic sperm injection; MII = Metaphase II; IVM: In Vitro Maturation

**Fig. 2:**
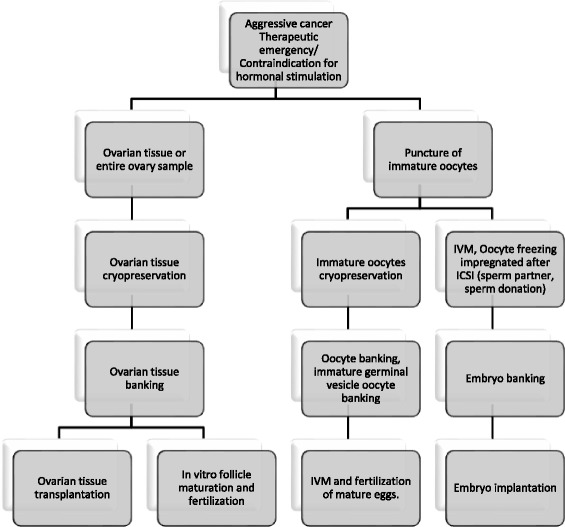
Current therapeutic strategy for female oncofertility when it is NOT possible to delay oncotherapy. ICSI: Intracytoplasmic sperm injection; MII = Metaphase II; IVM: In Vitro Maturation

Figures 1 and 2 show the fertility preservation healthcare circuit for female cancer patients commonly used worldwide. Unlike pubertal boys and men for whom sperm banking is an easy option, pubertal girls and young women face more difficulties when they hope to preserve their reproductive health. The female germ cell (only available in limited numbers) needs to be retrieved surgically mainly after hormonal stimulation, and will be at various levels of maturity depending on the length of the menstrual cycle. Currently, oocytes and embryo banking are standard of care for preserving fertility for reproductive-age cancer patients. The age, cancer site, timing and regimen of cancer treatment determine the optimal FP option. Excepting toxicity of treatments, the success of FP depends on the quality of the initial state of reproductive health of women before treatment. Little is known about the risk factors of various populations that should be taken into account.

Table 1 presents experimental levels, advantages, disadvantages and contraindications for FP options. While embryo cryopreservation is the first and most widely used option in the world, ovarian tissue banking is not universally available. Currently, there are relatively few medical centers with international experience in performing ovarian tissue banking: about 100 worldwide and including 23 in France [12]. An overview of Canadian practices [13] reported activities in terms of in vitro fertilization (IVF) across the country. They reported that 100.0% of IVF centres provide embryo cryopreservation, 82.4% provide oocyte freezing, 29.4% provide in vitro maturation services, and only 17.6% provide ovarian tissue preservation. In the U.S.A., the recourse to gestational carriers is an option for women who have undergone hysterectomy or severe damage to the uterus caused by oncology therapies.

**Table 1:** Experimental level, advantages, disadvantages and contraindications of FP options used in oncofertility around the world

FP option	Experimental level	Advantages	Disadvantages	Contraindication to FP technique
Embryo banking after puncture of mature oocytes	Standard method	Mature technology	Delay cancer treatment by 2-3 weeks Ethical and legal requirements Need for a partner with whom they wish to have a child	Presence of a CI to hormonal stimulation*
Embryo banking after puncture of immature oocytes	Experimental methods	Allows immediate cancer treatment	Ethical and legal requirements Need for a partner with whom they wish to have a child	
Mature oocyte cryopreservation	Experimental method Alternative to embryo cryopreservation for women who do not have a partner or do not want to use donated sperm	Legal property of the woman Better outcomes compared to IVM of cryopreserved immature oocytes	Delays cancer treatment by 2-3 weeks	Presence of a CI to hormonal stimulation *
Immature oocyte cryopreservation	Experimental method Women without partner or who do not want to use donated sperm	Allows immediate cancer treatment Legal property of the woman Less damage is caused by cryopreservation of immature oocytes than mature oocytes	Data on efficacy in cancer patients are not available	
Ovarian tissue transplantation	Highly experimental	Restoration of endocrine function	Invasive procedure Risk of reintroduction of aggressive cancer cells in some type of cancer **	Women older than 39 years
In vitro follicle maturation (IVM)	Highly experimental Alternative to tissue transplantation	Minimal risk for ovarian hyper stimulation syndrome	Technical difficulties	
Oophoropexy or Ovarian transposition	Experimental methods	Can be used for therapies requiring pelvic irradiation Ovarian protection Possible spontaneous pregnancy	No protection against chemotherapy or whole-body irradiation Carcinogenic risk	

*Contraindications (CI) to hormonal stimulation: prepubertal girls, hormone-responsive cancer, polycystic ovary syndrome

**Significantly elevated risk in patients with leukemia or ovarian tumor

Table 2 presents outcomes of FP options in cancer patients around the world. The total number of live births from the fertilization of mature cryopreserved oocytes exceeded 1000 in 2010 [5]. InVitro Maturation (IVM) was performed worldwide for thousands of conceptions leading to healthy babies. The success rate for ovarian tissue transplantation is still low [14, 15]. Rodriguez-Wallberg et al. [16] showed promising results in terms of recovery of fertility in Nordic countries that have performed ovarian tissue transplantation procedures.

**Table 2:** Outcomes of fertility preservation options in cancer patients in the world

FP option	Survey	Country	N	Mean age	Pregnancies	Live Births
Embryo freezing after ovarian stimulation	Barcroft et al., 2013, [30]	UK	42	31.9 ± 3.9	3 (1 twin)	3
Slow freezing embryos after ovarian stimulation	Lee et al., 2012, [31]	USA (NY)	151	36.2 ± 4.1 (LD group) * 34.9 ± 4.5 (HD group)	15 (LD group) 11 (HD group)	9 (LD group) 2 (LD group)
Oocyte vitrification	Garcia-Velasco et al., 2013 [32]	Spain	475	31.0 ± 5.1	2	1
Ovarian tissue tranplantation	Donnez et al., 2013, [33]	Belgium Denmark Spain	60	31.9 ± 5.1	18	12
Ovarian tissue transplantation	Dolmans et al., 2013, [34]	Belgium	476	23.0 ± 8.5	6	5

*N* number of patients. *LD group Low dose of FSH for ovarian stimulation, *HD group* High dose of FSH for ovarian stimulation

Currently, emerging technology is being developed: ovarian follicle culture in vitro, ovarian follicle transplantation, oogonial stem cells and in vitro activation of ovarian follicles. New FP options will continue to be developed.

### Organizational support of FP in cancer patients worldwide

For these patients in complex situations, health networks are essential to improve coordination of care, and the strengthening of this coordination is a major challenge to improve the performance of the health system. Functional support is required to mobilize all the necessary resources and guarantee an efficient healthcare circuit.

Two major international networks have been created in order to foster scientific exchange between countries and to standardize the FP healthcare circuit for cancer patients. In 2015 the International Network on Cancer, Infertility and Pregnancy (INCIP) incorporated the “Cancer in pregnancy” registry founded in 2005. The INCIP established an international registry on cancer during pregnancy and FP during cancer treatment and promotes research aiming to increase knowledge among healthcare workers and the public. The data collected include oncological, gynecological and obstetric data. All cancer types and treatment modalities are included. This international network seeks to work in close collaboration with existing networks notably the European Society of Gynaecological Oncology (ESGO) Fertility Preservation. The FertiPROTEKT network, founded in 2006, includes 100 centers in Germany, Austria and Switzerland to date. This network will now register their cases in the INCIP network. A German team [17] studied the impact of the creation of the FertiPROTEKT network and showed a significant increase in pregnancies within the first 2 years after breast cancer diagnosis in the period from 2010 to 2012 compared to the period from 2000 to 2002.

In the available literature, the governance of oncofertility is clearly identified in the public health policies of 4 countries: USA, Canada, Brazil and Australia-New Zealand. Table 3 presents networks and strategies of coverage worldwide. The French Society of Oncofertility was created as an association in 2013. The aim was to inform health professionals and the general public about the oncofertility field and to establish best oncofertility practices. In the development process, this association, located in Bondy, should be able to extend its task force to reach national scope. The European Society of Human Reproduction and Embryology (ESHRE) established in early 2017 a Special Interest Group focusing on FP, with the mission to support collaboration between European countries and with relevant professional bodies and societies. Health networks specialized in cancer research sometimes have a fertility preservation axis. In Europe, the ESGO has a FP task force responsible for promoting knowledge among healthcare workers and patients about oncofertility through national and international collaboration among specialist healthcare workers to promote research in order to develop new strategies for FP. In France, the regional network ONCOPACA has developed a regional Cancer and Fertility platform and a regional charter. FP is an objective of the French national Cancer Plan for the period 2014-2019, although there is no structured national network as yet. The Japanese Society for Fertility Preservation and the Korean Society for Fertility Preservation founded respectively in 2012 in Japan and 2013 in Korea, have created national FP networks including the oncofertility field. In low and middle countries, no structured preservation network was identified. In a study conducted in 2015, Mahajan [18] expressed the need for India to structure a multidisciplinary collaborative network to improve awareness of healthcare workers and FP service availability.

**Table 3:** Networks and strategies of coverage worldwide

Location	National Health networks	Role and Actions of the network	Tools	Other cooperating networks
USA	National Physicians Cooperative (NPC)	59 clinical sites across the US Information for patients, personalized management plan for patients Global fertility hotline Biological research Provides optimized protocols (including non biological parenting options)		• American society for reproductive Medicine (ASRM) • American Society of Clinical Oncology (ASCO)
	American Oncofertility Consortium	Produce guidelines Share informed consent document and consensus group decisions Implement standardized protocols Research into the societal, ethical and legal implications providing new perspectives on patient decision making	iSave Fertility app for physicians in English and Spanish Myoncofertility website for patients, parents and partners	
Canada	Oncofertility Referral Network	Platform that links patients, physicians and fertility clinics Resources for professionals Resources for patients		• The government agency Assisted Human Reproduction Canada • Canadian Cancer Society • Cancer Knowledge Network (Journal current oncology)
Brazil	Rede Brasileira de Oncofertilidade/ Brasilian Oncofertility Consortium	8 centers throughout Brazil Establish research projects and exchange on fertility options		
Australia/ New Zealand	Australasian Oncofertility Registry	Collect complete oncofertility data set from cancer and fertility centers Research projects		• Australasian Oncofertility Consumer group

National guidelines have been established in the U.S.A. by the American Society of Clinical Oncology (ASCO) [19] and the American Society for Reproductive Medicine (ASRM) [20] in 2013 and by the National Comprehensive Cancer Network (NCCN) in 2014 [21]. In Europe, guidelines have also been published by the ESHRE [22] and by the European Society of Medical Oncology (ESMO) 2013 [23]. There was also the publication of a report in France by the National Cancer Institute (INCa) and the Biomedicine Agency in 2013 [6] and in the U.K. by National Institute for Health and Clinical Excellence (NICE) in 2004. In Australia- New Zealand, an Australasian Oncofertility Consortium Charter was produced in 2015 by the Australasian Oncofertility Consortium. The International Society for Fertility Preservation established recommendations about this topic in June 2012 [24]. All these guidelines recommend universal access to FP facilities for young patients with cancer.

Table 4 presents an evaluation of knowledge, attitudes and practices of healthcare providers in the world. Forman et al. [25] concluded in a nationwide survey in the USA that 86% of oncologists consider it acceptable to sacrifice less than 5% reduction in disease-free survival to preserve fertility outcomes and 36% suggested that women would be willing to sacrifice more than 5%. Lack of time and lack of knowledge are identified as the main barriers to the initiation of FP discussion. Training of healthcare providers remains a challenge to meet the needs of quality of life of the patients. Kohlër et al. [26] showed that gender disparities in access to healthcare are strikingly against women. Table 5 presents an evaluation of healthcare circuits for patients worldwide.

**Table 4:** Evaluation of existing models- Knowledge, attitudes and practices of healthcare providers in the world

Location, Year	Authors, Reference	Study design	Main results
USA, 2011	Köhler et al., [26]	209 pediatric oncologists	83% of pediatric oncologists acknowledged that fertility threats to female patients are a major concern for them Only 12% reported that they refer at least 50% of female cancer patients to a fertility specialist prior to cancer treatment < 50% were aware of the ASCO recommendations published in 2006
USA, 2010	Forman et al., [25]	249 oncologists	95% routinely discussed a treatment’s impact on fertility: 93% for gynecologic oncologists vs 60% for other oncologists Although 82% have referred patients to reproductive endocrinologists, more than half rarely refer.
Canada, 2012	Yee et al. [35]	152 oncologists	45% did not know where to refer patients for female fertility preservation
Canada, 2013	Ronn and Holzer, [13]	All FP services available	63% of the responding non-IVF fertility centres do not provide any FP services, including consultations. 80% of the responding IVF fertility centres provided both consultations and FP services for women with cancer, with an additional 10% saying that they provide consultations only.
Iran, 2011	Ghorbani et al., [36]	30 specialists: 85% oncologists; 15% other specialists in cancer treatment	67% were attentive to the damaging effects of radiochemotherapy on fertility at the time of diagnosis 40% insisted that the FP topic should be brought up by patients themselves. Only 46% of the oncologists knew about FP techniques The greatest barrier to parental acceptance of FP for children was lack of information (41%)
France, 2013	Préaubert et al., [37]	225 French doctors from the PACA region	58% felt a lack information about indications and FP techniques 54% referred no patients to FP consultation over a period of 6 months
UK, 2008	Cannell [38]	84 Primary Care Trusts	46% did not provide patient information 33% did not commission facilities for embryo storage and 37% did not commission facilities for oocyte storage
UK, 2013	Adams, Hill and Watson, [39]	100 oncologists	87% expressed a need for information Only 38% reported routinely providing patients with written information 23% had never consulted any FP guidelines 1/3 did not usually refer patients to a specialist fertility service.
India, 2016	Mahajan et al. [9]	157 gynecologists	81% agreed with the ASCO recommendations 42% routinely discussed cancer impact on fertility 37% routinely discussed a treatment’s impact on fertility

**Table 5:** Evaluation of healthcare circuits for patients worldwide

Location	Authors, Date, Reference	Study design	Results
USA	Zebrack et al., 2004, [40]	32 childhood cancer survivors	Only 1/3 of patients had a discussion with the medical team on the risk of pregnancy during or after treatment.
USA	Salih et al., 2015, [41]	222 female childhood cancers survivors [≤21 years]	31% patients older than 13 years had decreased ovarian reserve or have premature ovarian failure 33 patients had reproductive counseling prior to treatment, only 2 had counseling during or after treatment 1 patient had oocryopreservation prior to initiation of chemotherapy.
USA	Kim et al., 2012, [42]	183 breast cancer patients	42% did not undergo FP treatment Women who had FP treatment were older, wealthier and had lower cancer stage
USA	Letourneau et al., 2012, [43]	1041 women with cancer	61% were counseled on the risk of cancer treatment for fertility 4% of women pursued FP Disparities in access to FP were observed based on educational level, ethnicity and sexual orientation
France	Huyghe et al., 2009, [44]	1000 cancer patients	20 to 30% would like to have more information on the potential risk of premature ovarian failure. 1/3 aged less than 50 years would have liked a fertility consultation before cancer treatment. 21% of women would definitely want to visit a reproductive health clinic in the next year.
France	Bouhnik et al., 2014, [45]	4349 cancer survivors 2 years after diagnosis	31.9% of women under 45 had a parental project 2/3 under 45 did not have FP discussion prior to initiation of treatment 2.2% of women under 45 had access to cryopreservation of gametes
Germany	Geue et al., 2013, [29]	149 cancer patients [18-45 years]	74% of patients wanted to have children at the time of diagnosis 50% of those who wanted a child needed supportive care concerning this issue 60% of the total sample had discussed fertility aspects with their oncologists and 20% with fertility specialists Men (56%) underwent fertility preservation more often than women (31%)
Sweden	Armuand et al., 2012, [46]	484 Patients(men and women)	48% of women reported that they received information about treatment impact on fertility. 14% reported that they received information about FP. Only 2% underwent FP Large gender disparities in access to FP care
UK	Corney and Swinglehurst, 2014, [47]	19 childless women aged below 45 withbreast cancer	Only half were given the opportunity to pursue assisted reproductive techniques prior to chemotherapy.

### International challenge of fertility preservation strategies

The paucity of nationwide referral networks is a challenge for the activities of international networks. Access to services remains limited, even in developed countries with specific health networks [13, 27] and to the best of our knowledge, no action plan has been published to develop the field of oncofertility in low and middle-income countries.

Financial costs for FP treatments are one of the major challenges thus far. In the U.S.A., in the state of Massachusetts, initial oocyte retrieval without medication costs 6000-12,000 USD and annual storage is around 440 USD [28]. In Canada, oocyte retrieval with intracytoplasmic sperm injection without medication is reported to cost 6000-8150 CAD, embryo cryopreservation ranged from 500 to 1200 CAD and oocyte retrieval and cryopreserved ranged from 2900 to 5400 CAD. The annual maintenance fee for cryopreservation and storage range from 200 to 800 CAD [13]. In 2013, Quebec was the only province in Canada that covered this cost burden. In Germany, cryopreservation of fertile eggs costs about $3400 and annual storage about $280 every year. In India, Mahajan et al. [9] showed in a study of Indian gynecologists that the second most common reason for not discussing the impact of cancer treatment on fertility was the cost of FP techniques (reason expressed by 34.5% of the gynecologists), after lack of available FP services in the city (35.9%). Geue et al. [29] indicated that these annual storage fees could easily become a psychological pressure for couples. In Belgium, the costs for GnRH analogues in not reimbursed. France is one of the few countries that offer women the assurance of financial reimbursement. Globally, financial reimbursement remains a thorny issue and FP imposes significant costs worldwide, restricting care access to women with low financial resources.

## Conclusion

By providing the international context of the organization of the oncofertility sector, this literature review aims to contribute to the development of new structures for the coordination of fertility preservation care in female cancer patients, particularly in low and middle-income countries. There is a structural gap in health care policies. Overall, the lack of information is demonstrated by the different internationally published surveys. Health care delivery should be organized in order to meet this need. The mobilization of skills acquired by collaboration through existing networks will make it possible to better structure this sector. To date, management strategies for oncofertility in the world are still fragile and unequal. To structure the oncofertility sector, a multidisciplinary project enabling teams to work together should be implemented, particularly in low and middle-income countries.

## Abbreviations

ASCO: American Society of Clinical Oncology
ASRM: American Society for Reproductive Medicine
ESGO: European Society of Gynaecological Oncology
ESHRE: European Society of Human Reproduction and Embryology
ESMO: European Society of Medical Oncology
FP: Fertility preservation
INCa: National Cancer Institute
INCIP: International Network on cancer, Infertility and Pregnancy
IVF: In vitro Fertilization
IVM: InVitro Maturation
NCCN: National Comprehensive Cancer Network
